# Gallstone Ileus: An Unusual Cause of Intestinal Obstruction

**DOI:** 10.7759/cureus.78364

**Published:** 2025-02-01

**Authors:** Siddharth Sankar Das, Suhasini Krishnan

**Affiliations:** 1 General Surgery, Dubai Hospital, Dubai, ARE; 2 Medicine, Dubai Academic Health Corporation, Dubai, ARE

**Keywords:** enterolithotomy, fistula, gallstone ileus, rigler's triad, small bowel obstruction

## Abstract

Gallstone ileus is a rare complication of gallstone disease, wherein a dislodged gallstone causes bowel obstruction by impaction of the intestines. Elderly patients and those with a history of gallstones are most commonly affected. Symptoms are usually vague and obscure, with patients reporting bloating, nausea and vomiting, early satiety, and constipation. To confirm the diagnosis, a contrast-enhanced computed tomography (CECT) scan is most commonly used, wherein Rigler’s triad of small bowel distention, aerobilia, and an ectopic gallstone may be seen, which prompts further management. Treatment would depend on the clinical circumstances, with either a conservative or surgical approach, although the latter is preferred in cases of intestinal obstruction. We have described a case of this very phenomenon, intending to bring awareness to its innocuous clinical presentation due to its potential fatality.

## Introduction

Gallstone ileus is a rare cause of bowel obstruction, characterized by the impaction of gallstones in the intestines [1,2]. Gallstone development is usually due to the crystallization and deposition of fats and minerals in the gallbladder. Although 80% of patients are asymptomatic, around 1%-2% experience symptoms requiring surgical intervention [3]. It makes up nearly 1%-4% of all cases of small bowel obstruction [4,5]. Considering the age group, it is most commonly observed in elderly female patients with concurrent comorbid conditions [1,3]. Symptoms most frequently experienced include abdominal pain, distension, nausea, and vomiting, which reflect underlying bowel obstruction [6]. Mortality has been reported to range from 12% to 27%, which can be avoided by a timely diagnosis and early recognition of symptoms [4]. Physicians need to be aware of the signs and symptoms of the condition due to its nonspecific clinical presentation [5]. Symptomatic cases warrant further radiological investigations to locate the dislodged stone(s). These investigations are beneficial in confirming the diagnosis and initiating treatment, which is usually surgical [1]. In this case report, we discuss a rare instance of this condition and have included a summary of existing and updated literature on the same.

## Case presentation

A 60-year-old female patient presented to the emergency department of the hospital with complaints of abdominal pain for the past four to five days associated with vomiting, weakness, and anorexia. She also reported a few episodes of constipation during this time. She did not have a fever. She has a history of allergy to aspirin and diclofenac.

Her past medical history was pertinent for gallstone disease, for which she was diagnosed two years prior. She mentioned having similar symptoms three months ago and was admitted to the hospital for a week and underwent conservative management. Other medical history was insignificant for any chronic illnesses and surgeries. 

The patient was conscious and cooperative during the physical examination, with stable vitals. The patient appeared well hydrated without signs of icterus or lymphadenopathy. The abdomen revealed distension and diffuse tenderness, which is more pronounced in the right upper quadrant and is associated with guarding and increased bowel sounds. No masses, organomegaly, or rebound tenderness was noted. Percussion revealed a tympanic abdomen with no fluid thrills or shifting dullness. Our provisional diagnosis at the time included acute pancreatitis and acute cholecystitis. 

**Figure 1:**
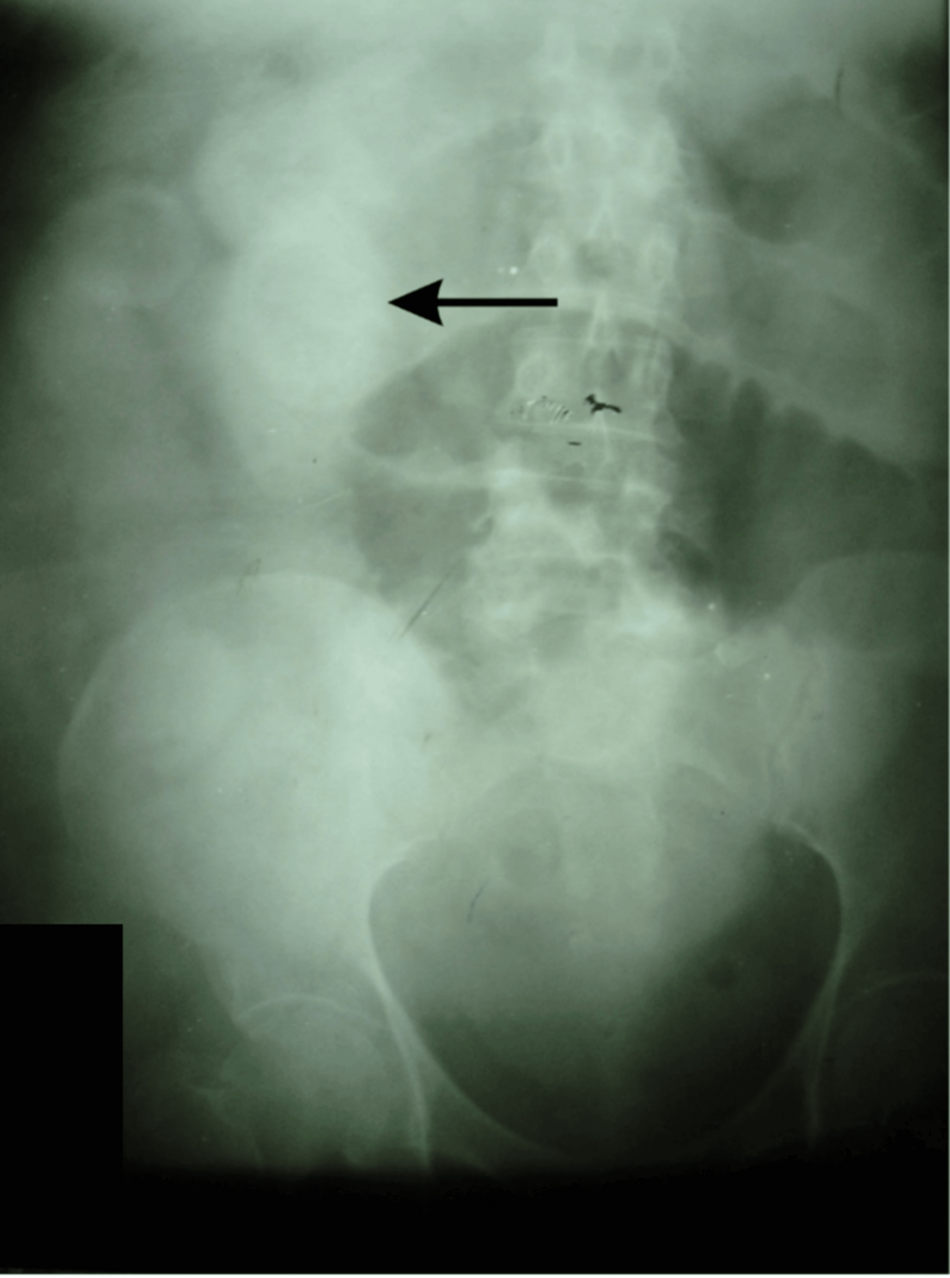
X-ray of the abdomen shows a calcified shadow on the right side of the abdomen.

**Figure 2:**
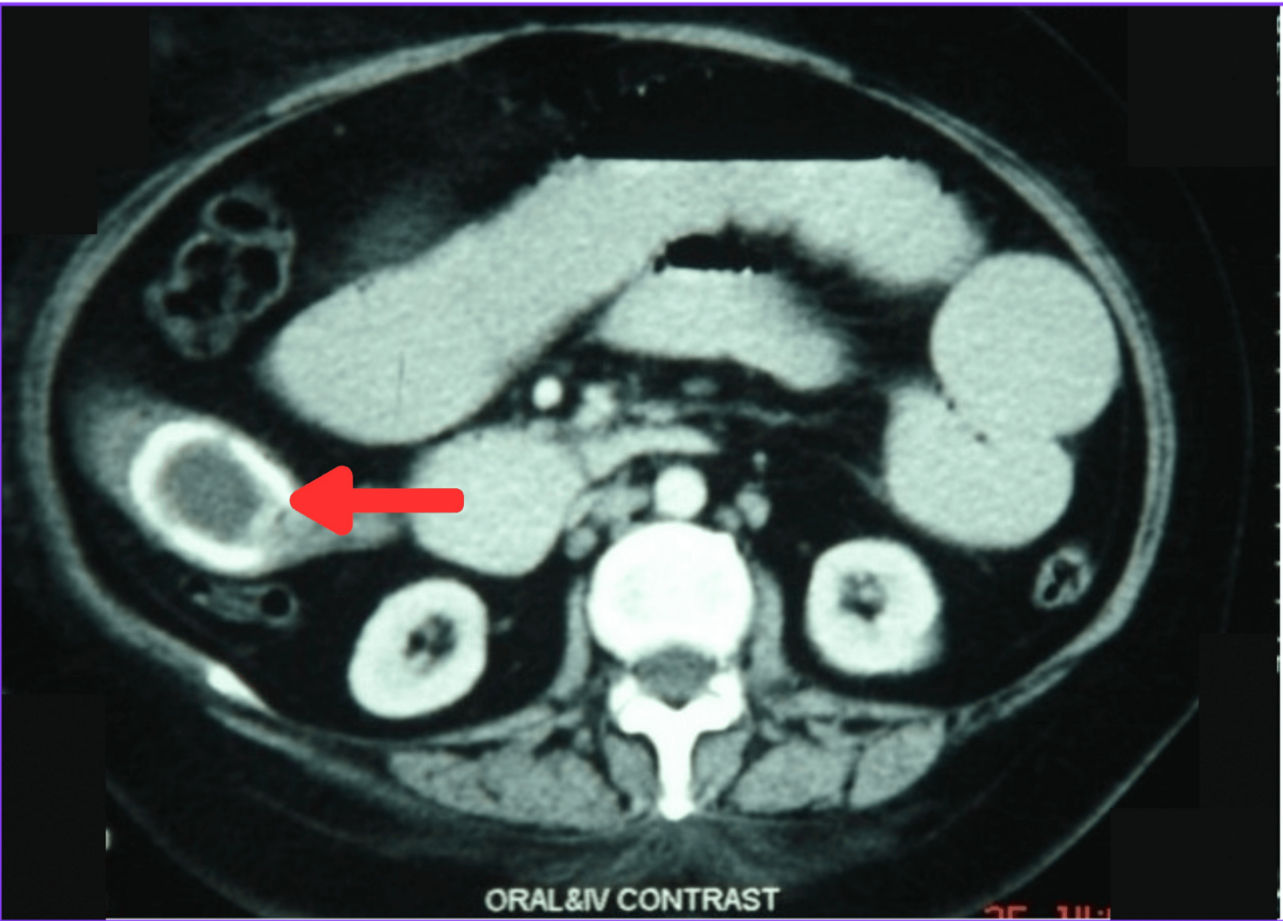
CT scan of the abdomen with contrast shows an impacted stone in the distal ileum.

Laboratory investigations revealed leukocytosis, an elevated Alkaline Phosphatase (ALP), and serum proteins, as seen in Table 1. Ultrasound done at the time of admission showed multiple gallbladder calculi, and once repeated after three days, showed a contracted gallbladder, some acoustic shadowing in the gallbladder fossa, and dilation of small bowel loops. Chest X-ray reported a raised right dome of the diaphragm, and abdominal X-ray showed two air-fluid levels in the central abdomen with diffuse haziness. A gastrografin study revealed a calcifying shadow in the right upper abdomen. Figure 1 reveals a calcified shadow in the right upper abdomen. A CT scan with contrast of the abdomen showed aerobilia, an ectopic gallstone in a small bowel loop with dilated proximal small bowel loops. Figure 2 shows an impacted stone in the distal ileum on a CT scan. Based on the investigations, a confirmatory diagnosis of gallstone ileus was made. 

**Table 1:** Laboratory parameters of the patient on admission.

Laboratory Results	Patient results (on admission)	Reference Values
Hemoglobin	10.5 g/dL	12.3-15.3 g/dL
Total Leukocyte Count	12 × 10^3^/µL	4.5-11.0 × 10^3^/µL
Alkaline phosphatase	214 IU/L	30 -120 IU/L
Serum proteins	5.0 g/dL	6.4 - 8.3 g/dL

**Figure 3:**
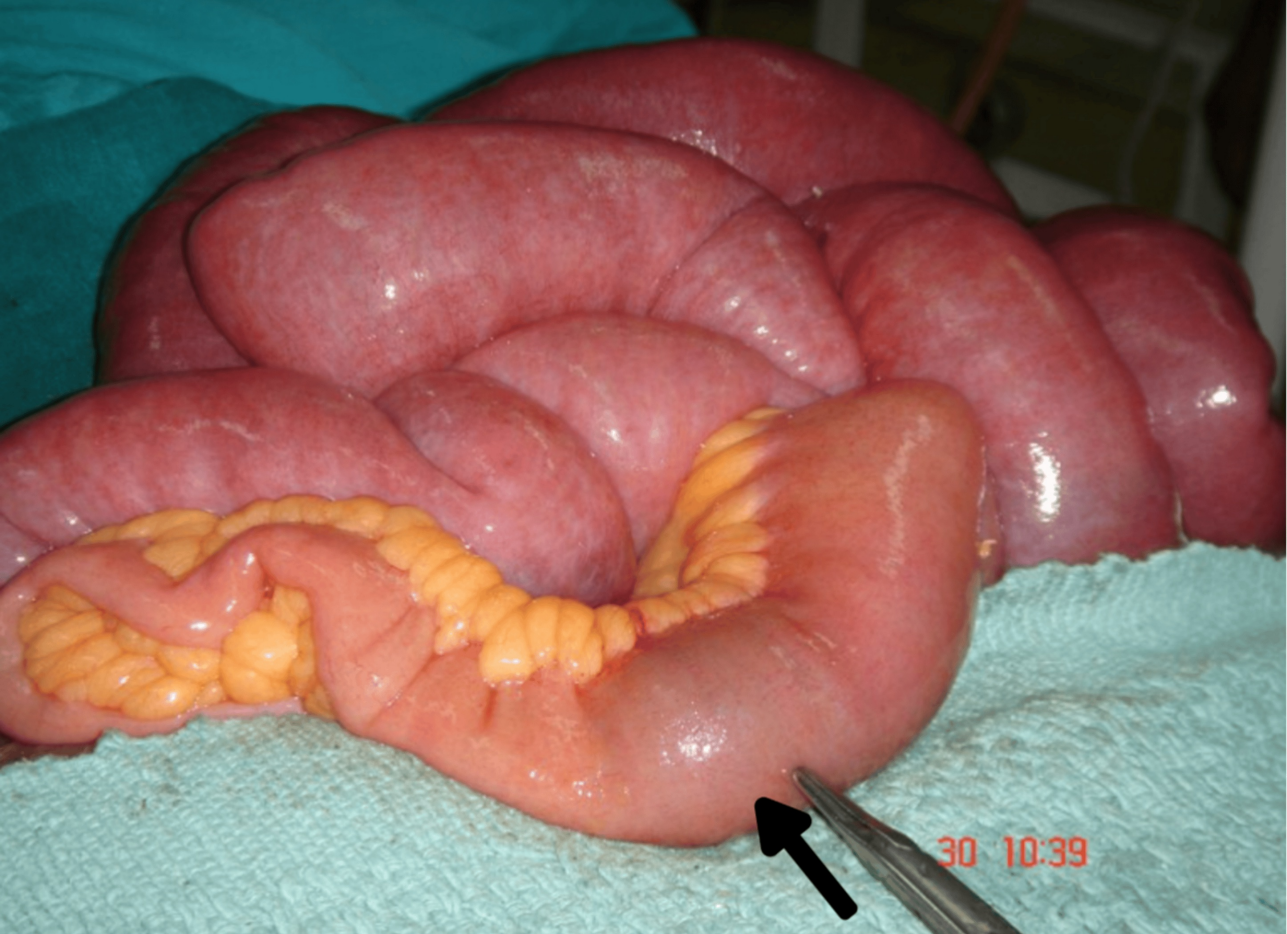
Impacted gallstone in the distal ileum with proximal dilated loops.

The patient subsequently underwent an exploratory laparotomy. Intraoperatively, small bowel loops were dilated up to approximately two feet proximal to the ileocecal junction; at this point, an enterolith of five centimeters was found. The distal bowel collapsed, and the calculus was impacted in the distal ileum, causing obstruction (Figure 3). In addition, dense adhesions were noted in the subhepatic region, obscuring the gall bladder completely with minimal ascitic fluid in the peritoneal cavity.

**Figure 4:**
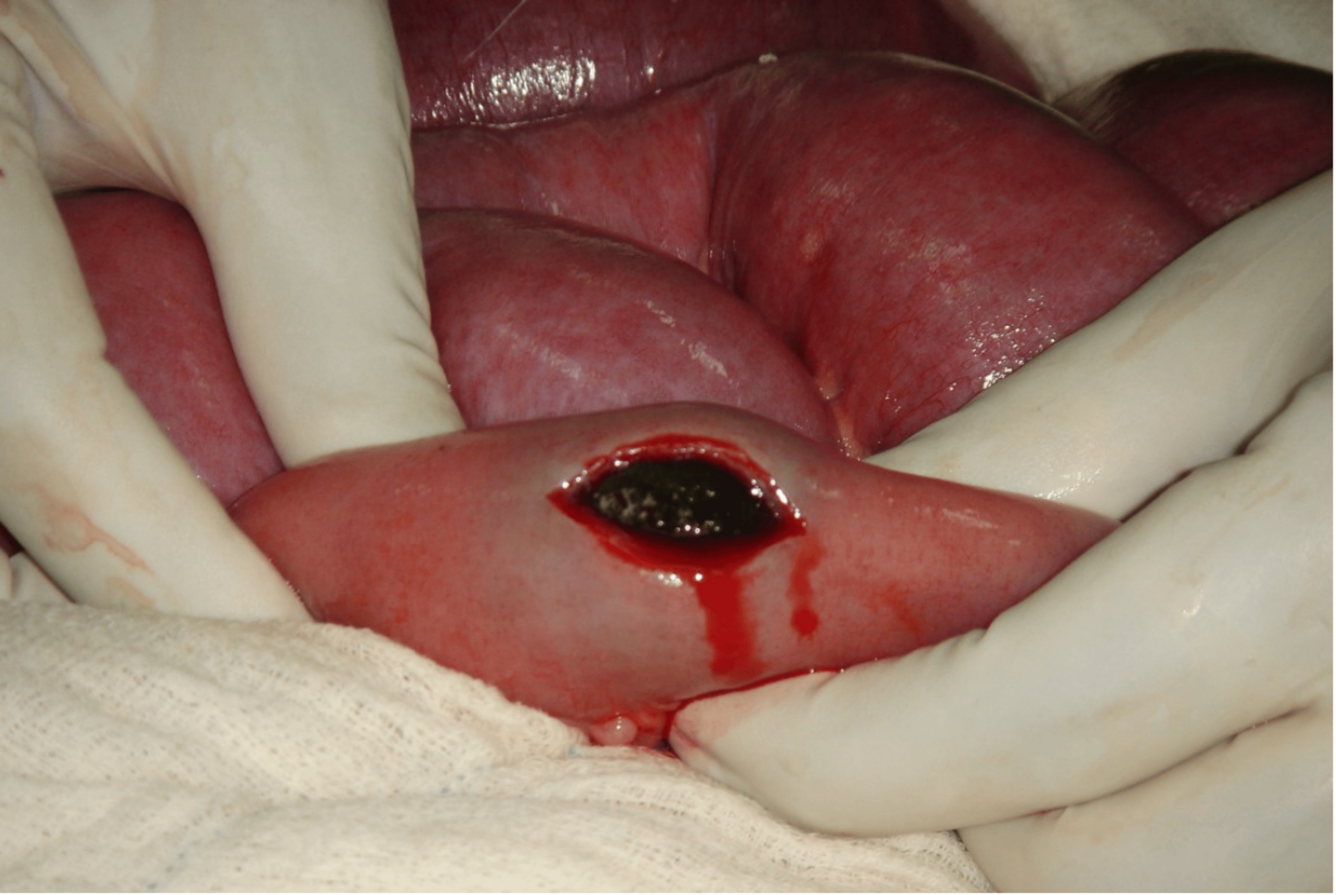
Enterotomy and removal of a gallstone from the distal ileum.

**Figure 5:**
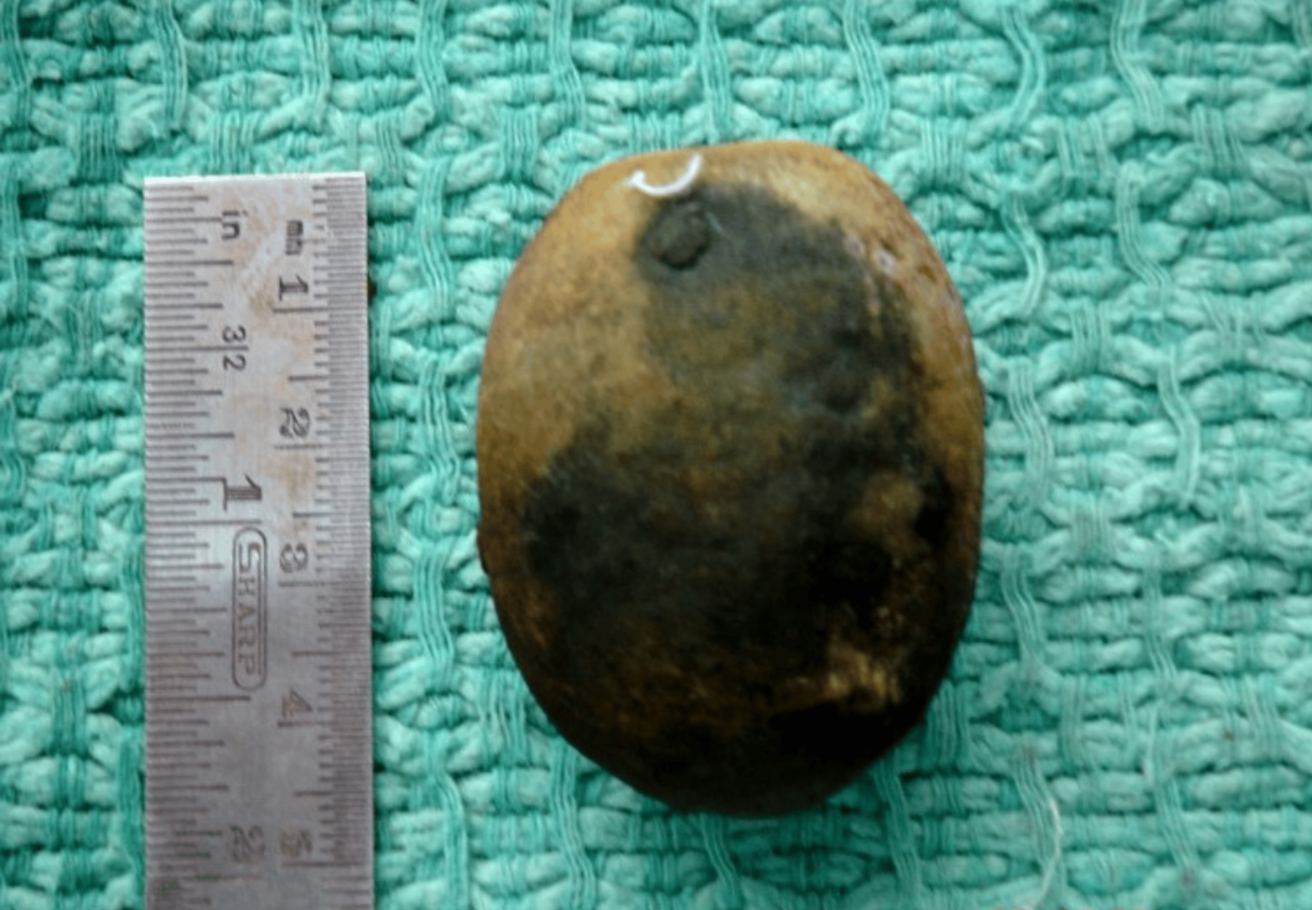
Removed gallstone following enterotomy.

Enterotomy was performed over the impacted section of the ileum, and a single gallbladder stone measuring approximately 5 cm was extracted (Figures 4-5). The enterotomy was closed transversely using 3-0 Vicryl in two layers, preventing small intestinal contamination. The complete small intestine was palpated manually for any other gallstone, but none other than the extracted stone was identified. Postoperatively, the patient recovered well, tolerated oral intake, regained peristalsis, and was discharged on the sixth postoperative day with oral antibiotics and analgesia.

## Discussion

Due to the proximity, it is through a biliary-enteric fistula that the gallstone travels from a necrotized gallbladder into the intestine [7]. This fistula is usually formed from erosion of the gallstone against the biliary wall [3,7]. However, there have been cases of gallstone ileus without a fistula [5]. The stone, once dislodged, may impact any section of the gastrointestinal tract [8]. Reisner and Cohen note that the terminal ileum and ileocecal valve are the most commonly affected gastrointestinal areas due to their small diameter and poor peristalsis [9]. Other regions not as frequently affected but may still be impacted are the jejunum, Treitz ligament, stomach, duodenum, and colon [6]. For obstruction to occur, the gallstone must be at least 2-2.5 cm in diameter [4,10].

The clinical presentation is variable and vague, making an accurate diagnosis challenging [11]. Before admission, patients may present with complaints of nausea, vomiting, abdominal pain, and distension, which may occur intermittently, as seen in our patient [12]. In some cases, at the time of obstruction, cholecystitis and jaundice may be present as well (10%-30% and less than 15%, respectively) [13].

Rigler’s triad is helpful in the identification and diagnosis of gallstone ileus using imaging modalities [13]. It includes features of small bowel obstruction with pneumonia (Gotta-Mentschler sign) and a gallstone that progressively changes location with serial imaging. A presence of two out of three indicates and confirms a diagnosis of gallstone ileus [3,4]. The most commonly employed imaging techniques include plain abdominal X-rays, ultrasound, and CT abdominal scans [11]. Ultrasonography of the abdomen can provide a clearer view of the fistula and pneumobilia [1]. CT scan of the abdomen is the most sensitive and specific (93% and 100%, respectively), and using an intravenous contrast can be particularly helpful [3,12].

Because low rates of spontaneous resolution have been reported, the aim of treatment is to remove the stone to prevent bowel obstruction [1,7]. The initial approach would be to decompress the bowel and start fluid resuscitation whilst keeping the patient nil per oral [3,6]. There have been cases of the condition being successfully managed with a conservative approach, such as lithotripsy, however most patients would require surgery [11,14]. There are three approaches to surgical management: an enterolithotomy, a one-step enterolithotomy, which includes cholecystectomy and repair of a fistula, and finally, a two-step enterolithotomy, which, after 4-6 weeks, is followed by cholecystectomy and closure of the fistula [1,12]. The first approach is most commonly employed; however, it has drawbacks. The open fistula could give rise to life-threatening complications such as cholangitis and sepsis [2,11]. Although quite rare, cases of recurrent gallstone ileus have been reported in approximately 5%-9% of patients [15]. There have also been reports of a higher morbidity rate in the one-step procedure despite a similar mortality rate across all approaches; this would reserve this option for a small patient population [1].

## Conclusions

It is important for clinicians to be mindful of gallstone ileus as part of the differential diagnoses in those at-risk patients, as if left undiagnosed can potentially be fatal. Patients can present with obscure symptoms and may or may not have a previous history of gallstones. Once a patient presents with those symptoms suggestive of subacute or intermittent bowel obstruction, clinical suspicion should be raised, the use of a CT scan can accurately confirm the diagnosis. Subsequently, it is up to the surgeon’s judgment to manage the case conservatively or, most preferably, surgically, employing one of the three aforementioned approaches.
